# ASCT2 (SLC1A5)-Deficient Mice Have Normal B-Cell Development, Proliferation, and Antibody Production

**DOI:** 10.3389/fimmu.2017.00549

**Published:** 2017-05-12

**Authors:** Etienne Masle-Farquhar, Angelika Bröer, Mehmet Yabas, Anselm Enders, Stefan Bröer

**Affiliations:** ^1^Research School of Biology, The Australian National University, Canberra, ACT, Australia; ^2^Department of Immunology and Infectious Disease, The John Curtin School of Medical Research, The Australian National University, Canberra, ACT, Australia

**Keywords:** SLC1A5, glutamine, B cells, glutaminolysis, ASCT2, glutamine uptake

## Abstract

SLC1A5 (solute carrier family 1, member 5) is a small neutral amino acid exchanger that is upregulated in rapidly proliferating lymphocytes but also in many primary human cancers. Furthermore, cancer cell lines have been shown to require SLC1A5 for their survival *in vitro*. One of SLC1A5’s primary substrates is the immunomodulatory amino acid glutamine, which plays an important role in multiple key processes, such as energy supply, macromolecular synthesis, nucleotide biosynthesis, redox homeostasis, and resistance against oxidative stress. These processes are also essential to immune cells, including neutrophils, macrophages, B and T lymphocytes. We show here that mice with a stop codon in *Slc1a5* have reduced glutamine uptake in activated lymphocytes and primary fibroblasts. B and T cell populations and maturation in resting mice were not affected by absence of SLC1A5. Antibody production in resting and immunized mice and the germinal center response to immunization were also found to be normal. SLC1A5 has been recently described as a novel target for the treatment of a variety of cancers, and our results indicate that inhibition of SLC1A5 in cancer therapy may be tolerated well by the immune system of cancer patients.

## Introduction

A well-known modification of metabolism in rapidly proliferating cells is the utilization of the amino acid glutamine in a linearized version of the tricarboxylic acid (TCA) cycle, termed glutaminolysis. As a result, many cancer cells display an oncogene-dependent “addiction to glutamine” *in vitro* (1–3). These metabolic pathways are not unique to tumor cells but are also used by certain normal cell populations. Glutamine utilization in proliferating B and T lymphocytes, for instance, can be very high and even comparable to that of glucose (4–7). Indeed, both glucose and glutamine are only partially oxidized in proliferating lymphocytes, similar to tumor cells.

In glutaminolysis, the amino acid glutamine is first deaminated to glutamate, then glutamate is further deaminated to α-ketoglutarate. The latter is converted *via* malate and oxaloacetate into aspartate, alanine, pyruvate, citrate, and acetyl-CoA (8, 9). A major advantage of this linear pathway is that it is less sensitive to depletion of its intermediate metabolites, in contrast to the TCA cycle that is compromised by removal of intermediates (9).

To proliferating cells, glutamine is therefore a major anaplerotic nutrient, as well as a major source of energy (8, 10–12). Indeed, expression of glutaminase (GLS) isozymes, the enzymes that catalyze deamination of glutamine to glutamate, correlates with growth rate and malignancy of certain cancer cells (13). In cancer, glutamine also sustains proliferative signaling *via* mammalian target of rapamycin (mTOR) activation and enables replicative immortality by suppressing senescence and resisting cell death (12).

While glutamine can be transported by a large number of solute carriers (SLC) (14, 15), a limited number of transporters dominate in rapidly proliferating cells. Along with SLC1A5, the leucine-preferring amino acid transporter 1 (LAT1 or SLC7A5) is highly upregulated in multiple cancers (1, 16, 17). One suggestion has been that SLC1A5-mediated import of glutamine provides an exchange substrate for the uptake of leucine (18) and other essential amino acids *via* LAT1, to meet metabolic demands and signal to mTOR (16). Coupled with the discovery that the glutaminolytic switch is promoted by oncogenes and inhibited by tumor suppressors, this has driven the search for SLC1A5 inhibitors (19–22). Indeed, blocking of SLC1A5 has been shown to diminish or prevent tumor cell proliferation in different cancers (23–28). In other cancer cells, SLC1A5 and LAT1 rather play the role of “amino acid harmonizers,” rapid hetero-exchangers that maintain the optimal mix of all 20 proteinogenic amino acids in the cytosol (9), while Na^+^-amino acid co-transporters of the SLC38 family mediate net glutamine uptake to sustain glutaminolysis and proliferation (9). For this second group of cancers, SLC1A5 antagonists or inactivating mutations of *SLC1A5* alone are insufficient to stop growth.

A significant concern in using amino acid transport inhibitors to treat cancer is the role of glutamine in the immune system. For example, B and T lymphocytes, which underpin the adaptive immune system, undergo phases of intense proliferation, both during their development and in order to fulfill their effector functions during immune responses. Because of the immune-modulatory effects of glutamine, and its requirement in strongly proliferating cells, the amino acid is crucial to normal development and effector functions of B and T cells (29–31).

In the hope of inhibiting SLC1A5 in cancer therapy, and because of the similar use of glutamine in cancer cells and the immune cells, understanding the role of SLC1A5 in the immune system is crucial. SLC1A5 has been shown to be required for rapid glutamine uptake during naïve T cell activation (32) because of its role in T cell receptor (TCR)-stimulated activation of mTORC1, which promotes cell growth and proliferation (33). Nakaya et al. (32) also found that SLC1A5-deficient mice had attenuated inflammatory T cell responses in experimental models of immunity and autoimmunity. In particular, the frequencies of CD4^+^ interferon γ-producing Th1 and IL-17-producing Th17 cells were significantly reduced. Furthermore, glutamine-deprived CD4^+^ T cells activated in the presence of cytokines that normally induce Th1 differentiation have been found to instead differentiate into Treg cells *in vitro* (34). Inhibiting SLC1A5 may therefore have adverse local and systemic effects on the immune system.

Here, we show that a C57BL/6 *Slc1a5* mutant mouse strain has normal B and T cell development and normal B cell effector functions, indicating that SLC1A5 could be targeted with limited impact on immune function.

## Materials and Methods

### Mice and Procedures

All experimental mice were housed in specific pathogen-free conditions at the Australian Phenomics Facility (APF) located at the Australian National University (ANU). All animal procedures for this study were approved by the ANU Animal Ethics and Experimentation Committee under protocol A2014/62. For all experiments, we used male and female mice between 7 and 14 weeks old.

The ENU28:008:SLC1A5 mouse strain was generated at the APF through ENU mutagenesis on a pure C57BL/6 background, as described previously (35). The strain was maintained by breeding heterozygous (*Slc1a5^+/−^*) mice with wild-type C57BL/6NcrlAnu mice or with heterozygous mice to produce homozygous (*Slc1a5^−/−^*) pups. ASD513:C57BL/6NcrlAnu mice are wild-type C57BL/6 mice originally obtained from Charles River and maintained by the ANU APF. *Rag1* KO (36) and *SW_HEL_* (37) mice have been previously described. ENU28:008:SLC1A5:Hc.Lc.Ly5a mice were generated by cross of ENU28:008:SLC1A5 mice and *SW_HEL_* mice. These mice will hereafter be referred to as *Slc1a5^+/+^* or *Slc1a5^−/−^SW_HEL_* mice.

Mouse primary immunization was performed as previously described (38) by i.p. injection of 50 µg alum-precipitated ARS-CGG (Biosearch) and 1 × 10^8^ heat- and formalin-inactivated *Bordetella pertussis* (BP) bacteria (Lee Laboratories) in 300 µL sterile PBS. Booster immunizations performed by i.p. injection of 50 µg ARS-CGG and 25 µg NP-Ficoll (Biosearch) in 300 µL sterile PBS. Antibody titers were measured by enzyme-linked immunosorbent assay as described previously (38).

### Glutamine Uptake

Glutamine uptake was assessed in *Slc1a5^+/+^* and *Slc1a5^−/−^* cells as described previously (39). Briefly, *Slc1a5^+/+^* and *Slc1a5^−/−^* splenocytes were cultured in RPMI 1640 (Sigma) supplemented with 10 mM HEPES pH 7.4 (Sigma-Aldrich), 0.1 mM non-essential amino acid solution (Gibco, Life Technologies), 1 mM sodium-pyruvate (Sigma-Aldrich), 55 µM 2-Mercaptoethanol (Life Technologies), 2mM l-glutamine, 100 U/mL penicillin-100 μg/mL streptomycin (Life Technologies), and 10% (v/v) heat-inactivated fetal calf serum (Life Technologies) (cRPMI) with 10 µg/mL LPS for 44–72 h to activate B cells and induce their proliferation. While this treatment also activates T cells, it mainly induces a proliferation of B cells and macrophages (40). Due to the large number of cells required for uptake experiments, splenocytes were used instead of purified B- and T-cells. The cell density of these suspensions was determined using an automated Vi-Cell XR counter (Beckman Coulter). Cells were then washed and resuspended at equal cellularity in Hanks (Na^+^) buffer (136.6 mM NaCl, 5.4 mM KCl, 0.44 mM KH_2_PO_4_, 0.5 mM MgCl_2_ × 6H_2_O, 0.41 mM MgSO_4_, 2.7 mM Na_2_HPO_4_ × 2H_2_O, 4 mM Hepes buffer, 100 mM CaCl_2_, 2 mM glucose, adjusted to pH 7.5 with NaOH), followed by different incubation times at 37°C with Hanks (Na^+^) buffer containing 0.1 mM glutamine and 0.1% v/v [^14^C] glutamine (1.85 Mbq/mL). Transport was terminated after the indicated times by centrifugation of the 100 µL cell suspension in an 0.4 mL polyethylene tube (Sigma) containing 65 µL silicone oil (density 1.03 g/mL to strip the cells from their extracellular medium) on top of 30 µL 20% perchloric acid. Cells were then pelleted and resuspended in 750 µL ddH_2_O, followed by transfer of 500 µL homogenate into scintillation vials, and addition of 2.5 mL scintillation fluid. Radioactivity was measured by liquid scintillation counting. An aliquot of each B cell suspension was used to determine the protein content of each sample by Bradford assay (Sigma).

### Flow Cytometry and FACS Analysis

Flow cytometric analyses were performed as previously described (41). Briefly, mice were euthanized using cervical dislocation and the organs collected in PBS + 2% heat-inactivated FBS (FACS buffer). The spleen and thymus were forced through a 70-µm cell strainer to create a homogenous single-cell suspension in FACS buffer. Bone marrow cells were isolated from mouse hind limbs by flushing the femur and tibia with FACS buffer followed by passing through a 70-µm cell strainer. Peritoneal cavity cells were collected by injection of 3 mL FACS buffer into the peritoneal cavity followed by redrawing. Cell numbers in the resulting cell suspensions were determined using an automated Vi-Cell XR counter (Beckman Coulter). Equal cell numbers—depending on organ and experiment between 1 and 2 × 10^6^ cells per sample—were stained with antibodies at predetermined optimal dilutions in FACS buffer at 4°C, followed by analysis on an LSRII or an LSRFortessa (BD). Hen egg lysozyme (HEL)-binding B cells were detected by incubating the cells with HEL (Sigma) followed by staining with fluorescently labeled HyHEL9 antibody, which recognize a different epitope on HEL than the transgenic BCR. The HyHEL9 antibody was a kind gift of Professor Robert Brink from the Garvan Institute, Sydney. Conjugation to Alexa Fluor 647 was done in house using the Alexa Fluor 647 Antibody labeling kit (Thermo Fisher Scientific) according to the manufacturer’s instructions. All other antibodies were purchased from Biolegend, eBioscience, or BD Pharmingen and titrated in house for their optimal dilution. Compensation for multicolor stains and data acquisition were performed using the FACSDiva software (BD), and data analysis was performed using the FlowJo software (Tree Star).

### B Cell Proliferation Assays

B cells were purified using a Pan B cell Isolation Kit (Miltenyi Biotec) followed by negative sorting using the DepleteS protocol of the autoMACS Pro Separator (Miltenyi Biotec). 10 × 10^6^ to 30 × 10^6^ B cells were resuspended in 1 mL cRPMI l. The B cells were labeled with Cell Trace Violet (CTV, Invitrogen) by addition of CTV to obtain a final concentration of 12.5 µM followed by immediate vortexing to ensure rapid and homogeneous labeling of cells. Cells were incubated for 20 min at 37°C in the dark, and the CTV labeling reaction was terminated by addition of 12 mL cRPMI and two successive washes with cRPMI. Finally, cells were added to individual wells of a 96-well round-bottom plate at a concentration of 2 × 10^6^ cells/mL and incubated for 10 min at 37°C to allow the CTV to undergo acetate hydrolysis. Finally, 100 µL of cRPMI or cRPMI containing different stimuli were added to each well resulting in a final cell concentration of 1 × 10^6^ cells/mL. The following stimulus conditions were used: unstimulated (cRPMI only); anti-IgM (1 or 10 µg/mL) combined with anti-CD40 (10 µg/mL) or lipopolysaccharide (LPS; 10 µg/mL). Cells were incubated for 36–72 h in a humidified incubator at 37°C in 5% carbon dioxide (CO_2_) before staining.

### Isolation and Culture of Primary Mouse Fibroblasts

Preparation of fibroblasts from mouse tail-tips was carried out as described by Takahashi et al. (42) without modification. Glutamine uptake in primary cultures was measured as described by Heckel et al. (43).

### Generation of Bone Marrow Chimeras

Bone marrow chimeras were generated as described previously (38). In short, C57BL/6 *Rag1* knockout mice were sublethally irradiated with 4.5 Gy followed by intravenous injection of 2 million bone marrow cells from either wild-type or *Slc1a5^−/−^* mutant mice or with a mix consisting of 50% wild-type and 50% *Slc1a5^−/−^* bone marrow cells (100 and 50–50% bone marrow chimeras, respectively). In the case of mixed bone marrow cells, the co-transferred wild-type cells were CD45.1 allotype marked to allow differentiation from the CD45.2 allotype *Slc1a5^−/−^* cells.

### Statistical Analysis

All data were statistically analyzed using Graph Pad Prism 5.0f for Mac OS X. Comparison between two groups was done using an unpaired *t*-test. For the proliferation data shown in Figure 2, we used ANOVA with Sidak analysis for multiple comparisons.

## Results

Sequencing of *Slc1a5* mutant mice revealed a 1225T>A substitution mutation in *Slc1a5* resulting in a premature stop codon at amino acid 224 of the 555 amino acid SLC1A5 protein.

**Figure 1:**
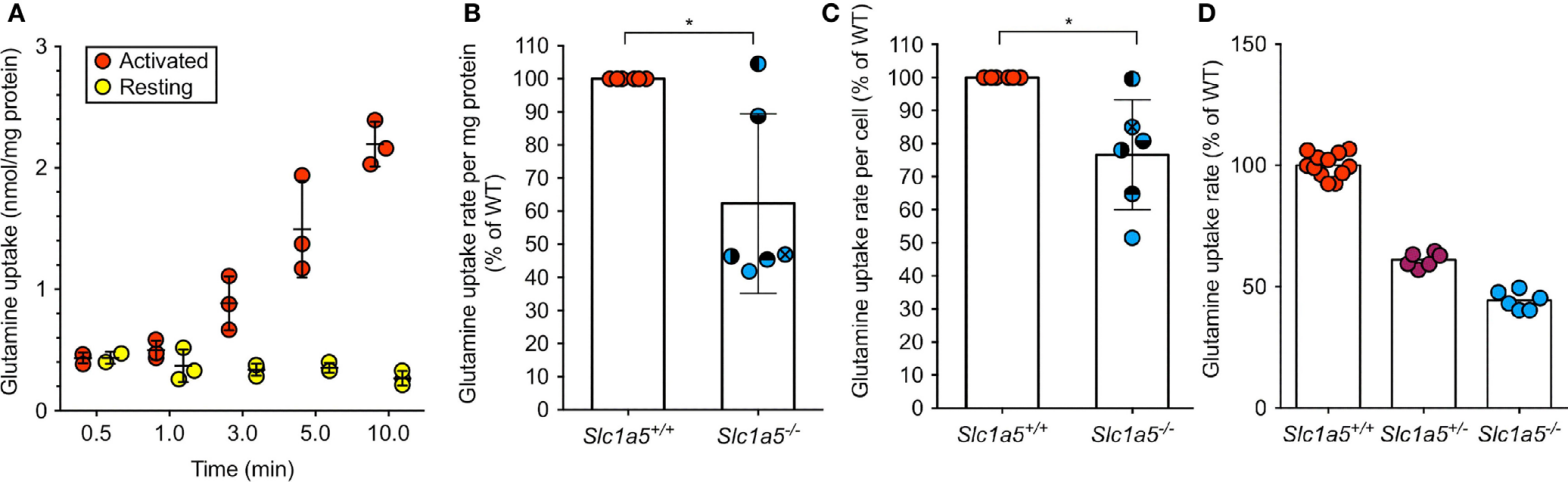
**Abrogated SLC1A5 protein expression in Slc1a5^−/−^ mice results in reduced glutamine uptake**. **(A)** Glutamine uptake over time in resting B cells or after 48 h LPS activation. **(B,C)** Glutamine uptake in LPS-activated B cells from *Slc1a5^+/+^* and *Slc1a5^−/−^* mice normalized to mg protein **(B)** or cell number **(C)**. Data pooled from six independent experiments. To compare data in panels **(B,C)**, each experiment has individual symbols. **(D)** Glutamine uptake in primary mouse fibroblasts isolated from *Slc1a5^+/+^, Slc1a5^+/−^*, and *Slc1a5^−/−^* mice. Data from two experiments with six technical replicates (*Slc1a5^+/+^*) or one experiment with six technical replicates. All data normalized to the average of *Slc1a5^+/+^* cells analyzed in the same experiment. For data in panels **(B,C)**, statistical analysis was done using an unpaired *t*-test (*p < 0.05). Error bars show SD.

To determine the impact of a *Slc1a5* null allele, glutamine uptake assays were performed. Glutamine uptake in resting wild-type B cells was hardly detectable, but was found to increase drastically upon activation by LPS (Figure 1A). Thus, lack of SLC1A5 expression was assessed in LPS-activated B cells, where the rate of glutamine uptake was reduced to 62 and 77% of glutamine uptake in wild-type B cells, depending on whether transport was normalized to protein content or cell number, respectively (Figures 1B,C). This result is consistent with a significant, but not exclusive role of SLC1A5 in glutamine uptake in activated B-cells. To confirm functional SLC1A5 inactivation in a well-characterized cell type, we cultivated primary fibroblasts from tails of wild-type, SLC1A5^+/−^ and SLC1A5*^−/−^* mice. As shown in Figure 1D, glutamine uptake was significantly reduced upon partial and complete inactivation of SLC1A5.

**Figure 2:**
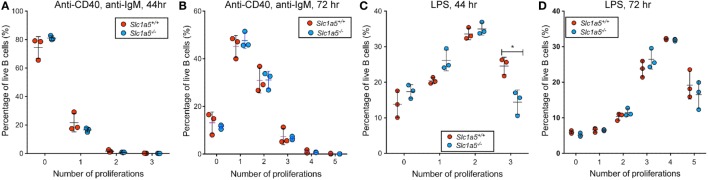
**A transient delay in B cell proliferation in response to strong stimulation**. Lymphocytes in single cell suspension were cultured in cRPMI supplemented with 10 μg/mL anti-CD40 and anti-IgM LPS **(A,B)** or LPS **(C,D)** for 48 h **(A,C)** or 72 h **(B,D)**. Proliferation was measured by halving in Cell Trace Violet (CTV) signal intensity upon each cell division. The *Y*-axis shows the percentage of B cells that have undergone the number of cell divisions shown on the *X*-axis. *n* = 3 biological replicates. Data are representative of results obtained for *n* = 6 biological replicates. Statistical analysis was done using ANOVA with a pairwise comparison between WT and *Slc1a5* mutant cells that have undergone the same number of cell divisions. Except where indicated, no significant difference was found (*p < 0.05).

Given the strong increase in consumption of glutamine upon lymphocyte activation (4, 5), and the well-established importance of glutamine in metabolic pathways necessary for B and T cell proliferation (8, 30, 31), the proliferative capacity of wild-type and *Slc1a5^−/−^* B cells was assessed *in vitro*. Following TLR4-mediated or BCR- and CD40-mediated stimulation of purified B cells, cell proliferation was assessed based on dilution in fluorescence intensity of the intracellular dye CTV. While no difference was observed following treatment with soluble F(ab′)_2_ fragments of anti-IgM (hereafter referred to as anti-IgM) and anti-CD40 agonists after 44 or 72 h (Figures 2A,B), *Slc1a5^−/−^* B cells were found to have slower division kinetics in response to 44 h LPS stimulation (Figure 2C). The lack of any difference in proliferation after stimulation with anti-IgM and anti-CD40 agonists was likely due to weaker B cell proliferation in response to this stimulus relative to that in response to LPS stimulation. Thus, SLC1A5 deficiency decreases glutamine uptake rate in lymphocytes, but it only becomes limiting for B cell proliferation in response to strong stimulation.

Furthermore, the difference in proliferation of *Slc1a5^−/−^* and wild-type B cells was no longer observed after 72 h stimulation (Figure 2D), indicating that the delay in proliferation in response to LPS stimulation is only transient. This may be due to a rescue in glutamine uptake in *Slc1a5^−/−^* B cells by upregulation of redundant transporters—e.g., SLC38A2, which is known to be upregulated by stress such as amino acid depletion—or by the use of alternative metabolic pathways over time.

**Figure 3:**
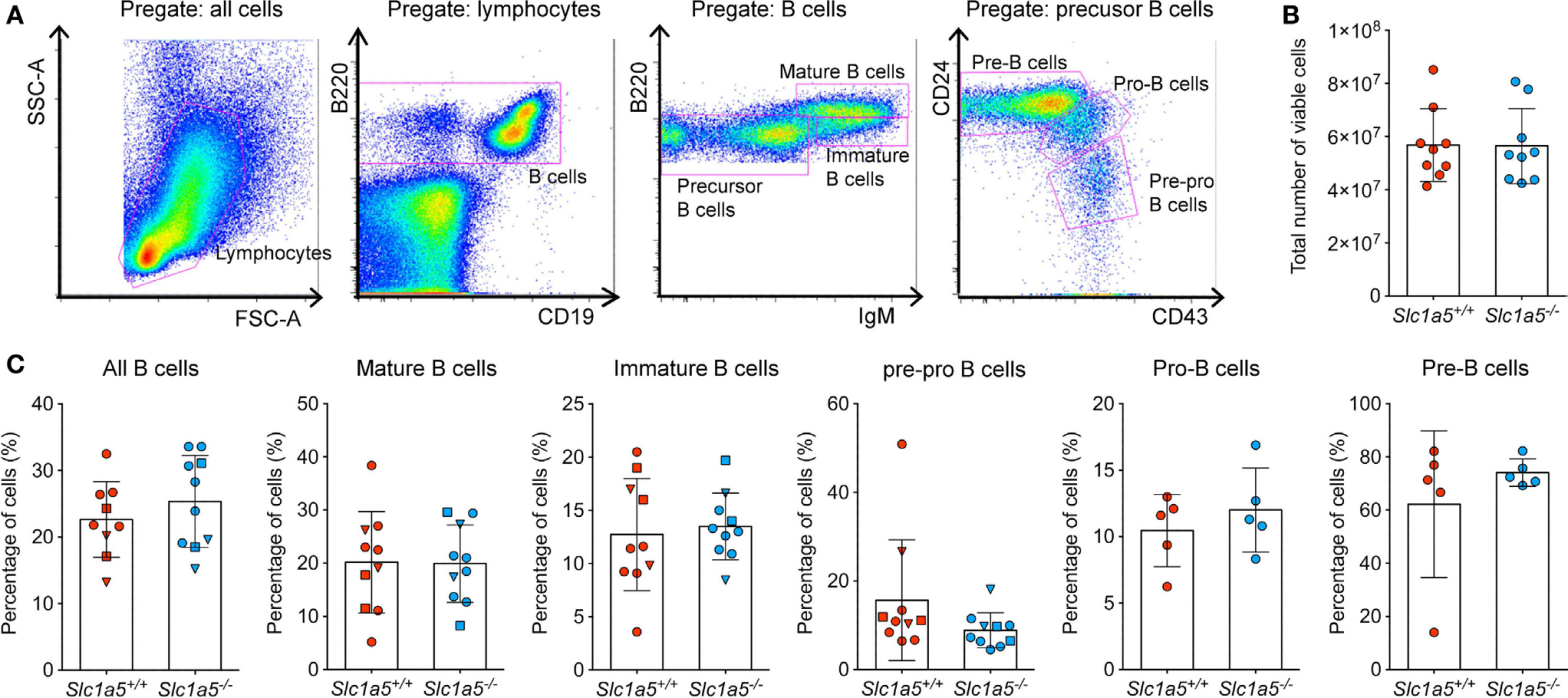
**B cell development is unchanged in the bone marrow of *Slc1a5^−/−^* mice**. **(A)** Representative gating strategy, **(B)** total number of viable cells, and **(C)** percentages of B cells at different developmental stages in the bone marrow. Data were pooled from three different experiments with 2–6 mice per group and experiment. Each experiment is shown by a different symbol. For panels **(B,C)**, statistical analysis between *Slc1a5^+/+^* and *Slc1a5^−/−^* mice was done using a Student’s *t*-test, no significant differences were found. Error bars show SD.

**Figure 4:**
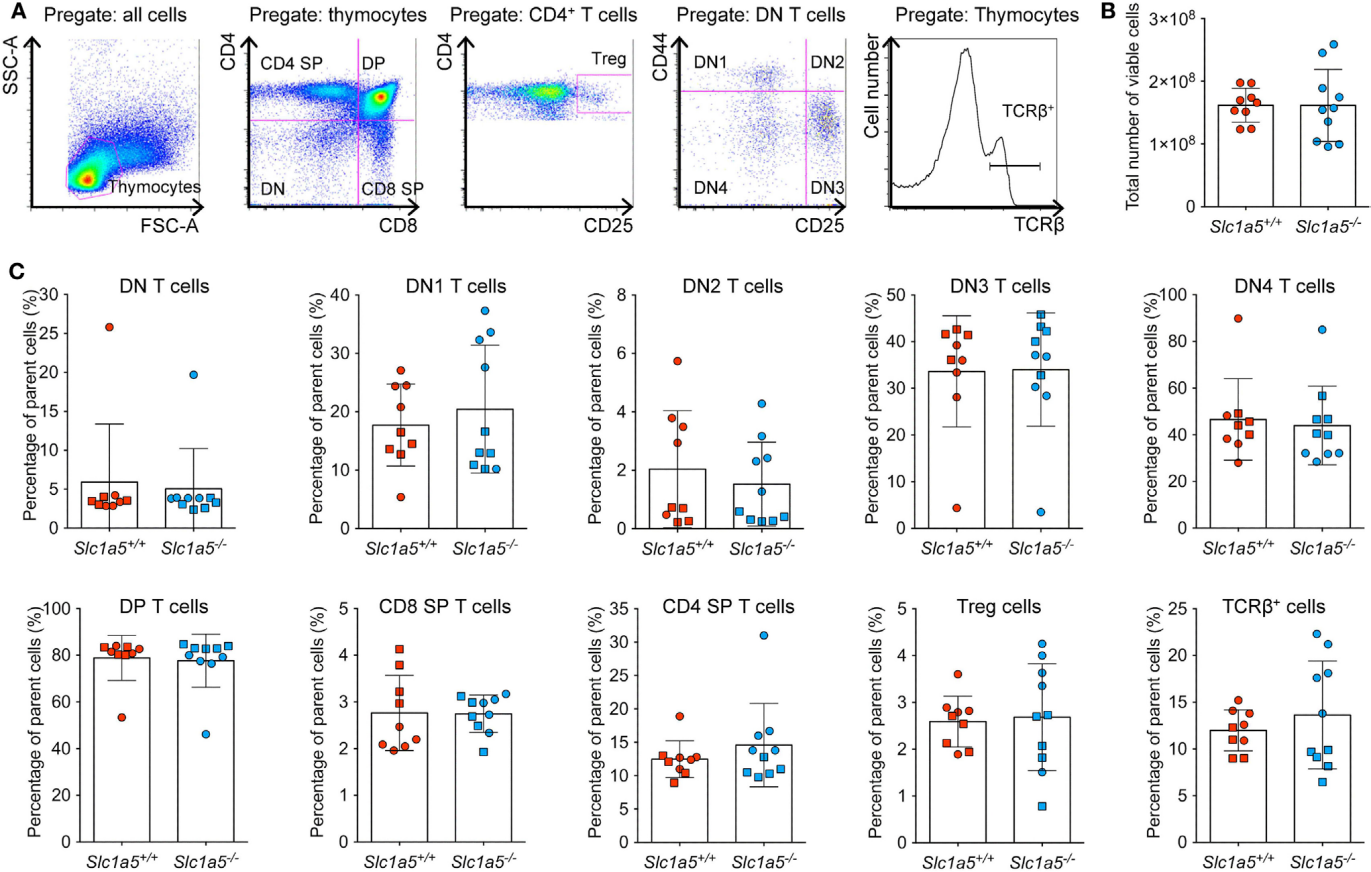
**Normal T cell development in the thymus of *Slc1a5^−/−^* mice**. **(A)** Representative gating strategy, **(B)** number of viable cells, and **(C)** percentages of T cells at different developmental stages in the thymus. Data were pooled from two separate experiments with 4–5 mice per group and experiment with each experimental group shown by a different symbol. For panels **(B,C)**, statistical analysis between *Slc1a5^+/+^* and *Slc1a5^−/−^* mice was done using an unpaired *t*-test, no significant differences were found. Error bars show SD.

**Figure 5:**
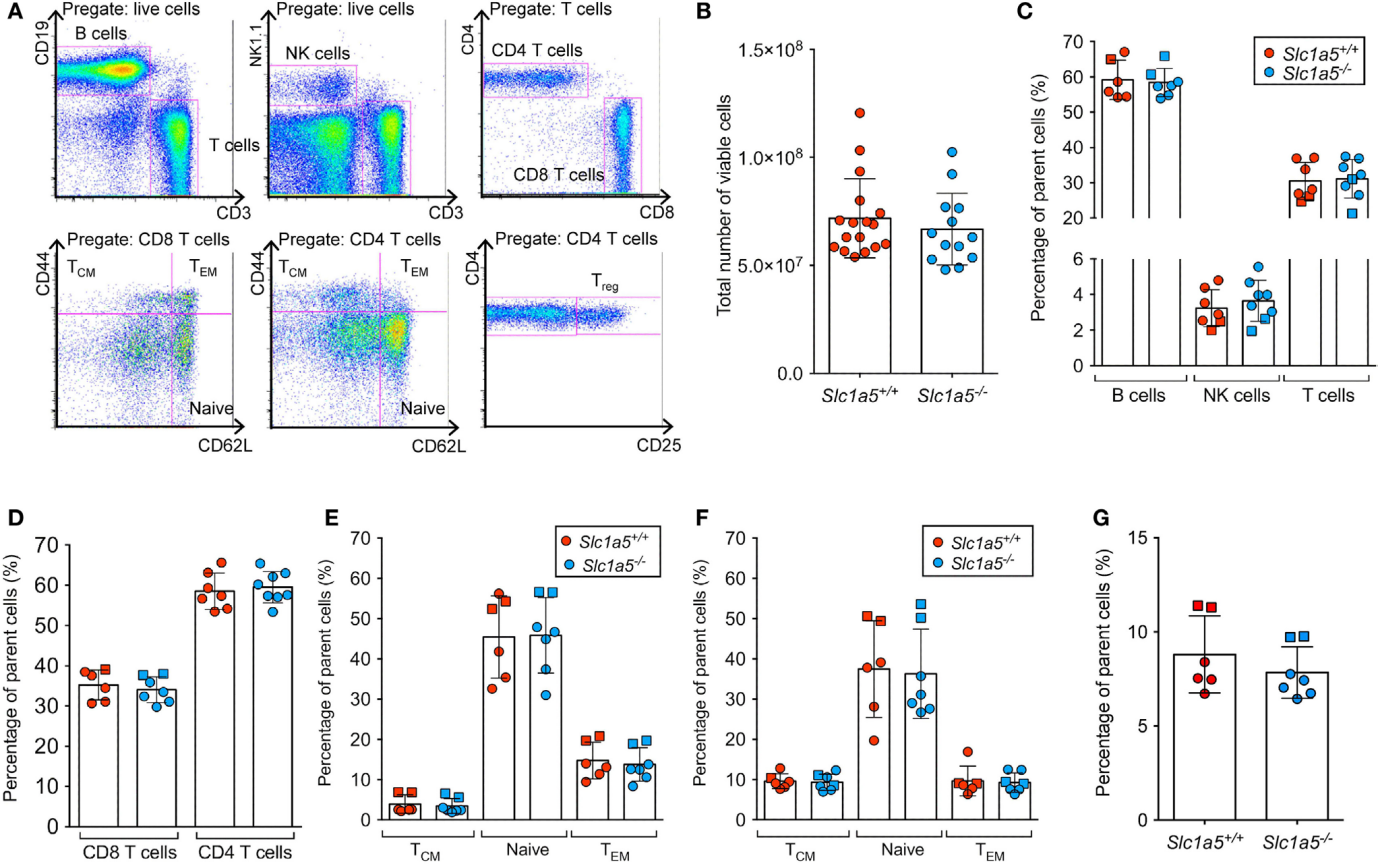
**No differences in T cell populations in the spleen of Slc1a5^−/−^ mice**. Representative gating strategy for T cell populations in the spleen **(A)**, total number of live splenocytes **(B)**, relative percentages of the main splenocyte populations **(C)**, relative percentages of CD4 and CD8 T cells **(D)**, of naïve, central memory, and effector memory CD4 **(E)** and CD8 **(F)** T cells, and of T regulatory cells **(G)**. Data were pooled from two separate experiments with two and five mice per group, different experiments are shown by the use of different symbols (squares or circles). Error bars show SD.

**Figure 6:**
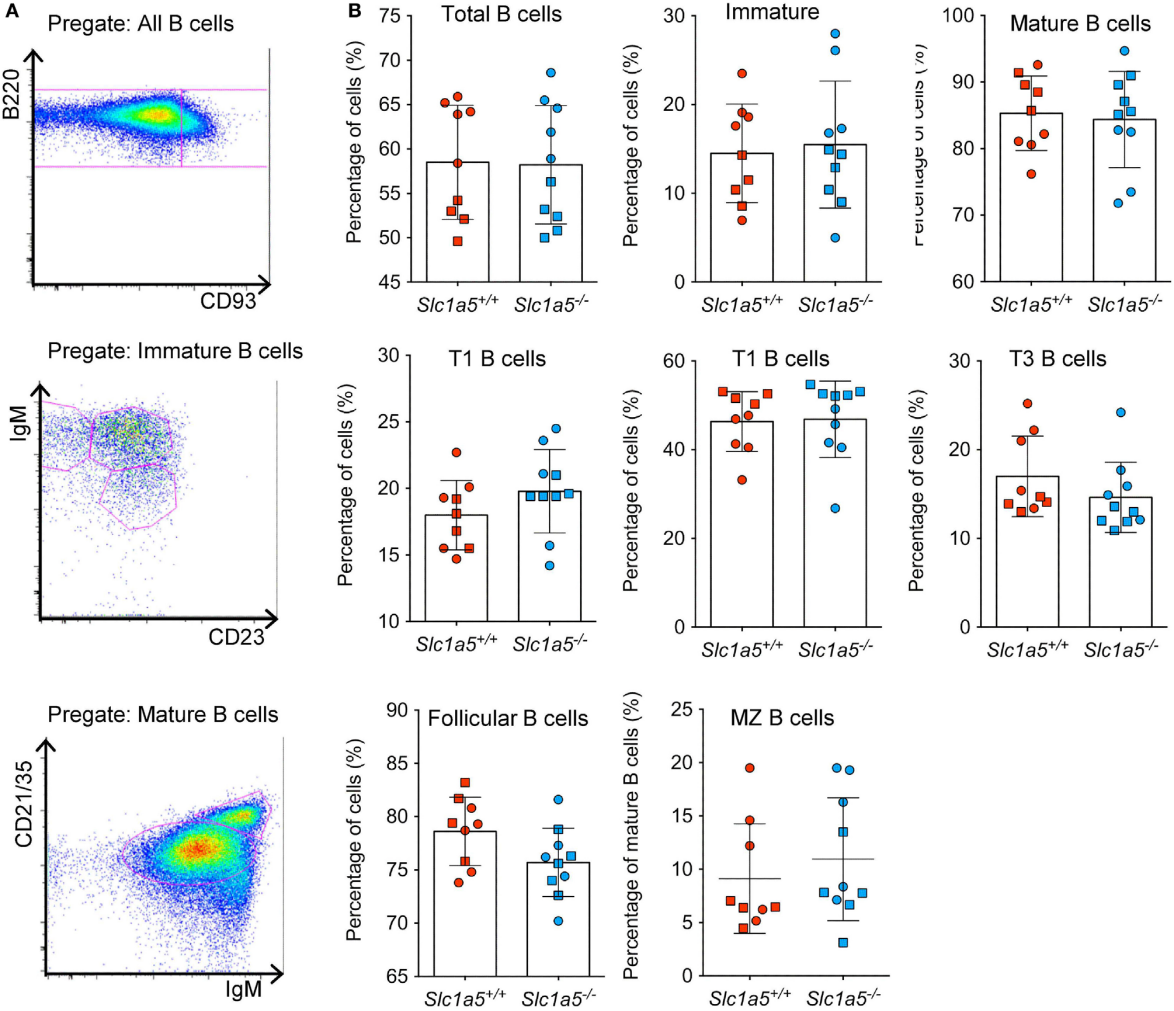
**Normal B cell development in the spleen of Slc1a5^−/−^ mice**. Representative gating strategy **(A)**, and percentages of B cells at different developmental stages **(B)** in the spleen. Data were pooled from two separate experiments with 4–5 mice per group and experiment. Each experiment is shown by a separate symbol. Statistical analysis between *Slc1a5^+/+^* and *Slc1a5^−/−^* mice was done using an unpaired *t*-test, no significant differences were found. Error bars show SD.

Stimulation of immune cell populations *in vitro* replicates the *in vivo* situation only partially. Therefore, we examined the effect of SLC1A5 deficiency on development and maturation of both B and T cells *in vivo*. Both cell types undergo several phases of strong proliferation during maturation, which may be affected by SLC1A5 deficiency. Flow cytometric analysis (Figure 3A) revealed that the total cell number in the bone marrow (Figure 3B) was not affected by SLC1A5 deficiency. Similarly, the frequencies of B cell subpopulations in the bone marrow (Figure 3C) and developing T cells in the thymus (Figure 4) were not affected by the lack of SLC1A5. Similarly, the B2 cell populations in the spleen (Figure 5) and the B1 cell populations in the peritoneal cavity (not shown) of wild-type and *Slc1a5^−/−^* mice were not significantly different, nor were the T cell populations in the spleen (Figure 6). Thus, global loss of SLC1A5 expression had no significant effect on the maintenance of cell populations of the adaptive immune system.

To detect subtle competitive disadvantages of *Slc1a5^−/−^* cells, B and T cell development and maturation was further investigated in the competitive *in vivo* environment of 100% and mixed bone marrow chimeras. Peripheral blood of 100% wild-type or *Slc1a5^−/−^* hematopoietic stem cell (HSC) recipients was collected 4 and 6 weeks post-irradiation, and the cell subsets present were found in equal proportions (data not shown). Moreover, lymphoid organs of both 100 and 50–50% bone marrow chimeras were harvested 7 weeks post-irradiation. No significant differences in B and T cell development and maturation were detected (Figure S1 in Supplementary Material).

**Figure 7:**
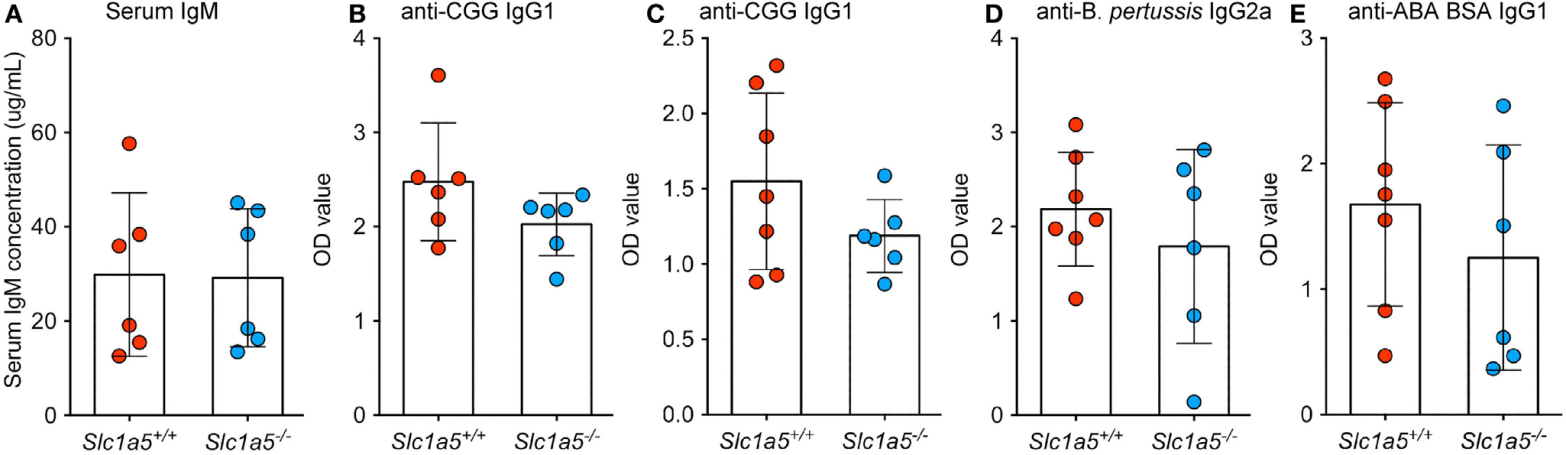
**No differences in antibody responses to immunization between *Slc1a5^+/+^* and *Slc1a5^−/−^* mice**. Serum IgM concentration in un-immunized mice **(A)**, relative concentration of IgG1 antibodies to CGG 2 weeks **(B)** and 4 weeks **(C)** post-primary immunization, of IgG2a antibodies to *Bordetella pertussis* **(D)** and IgG1 antibodies to ABA BSA **(E)** 4 weeks post-primary immunization. Statistical analysis between *Slc1a5^+/+^* and *Slc1a5^−/−^* mice was done using an unpaired *t*-test, no significant differences were found. Error bars show SD.

**Figure 8:**
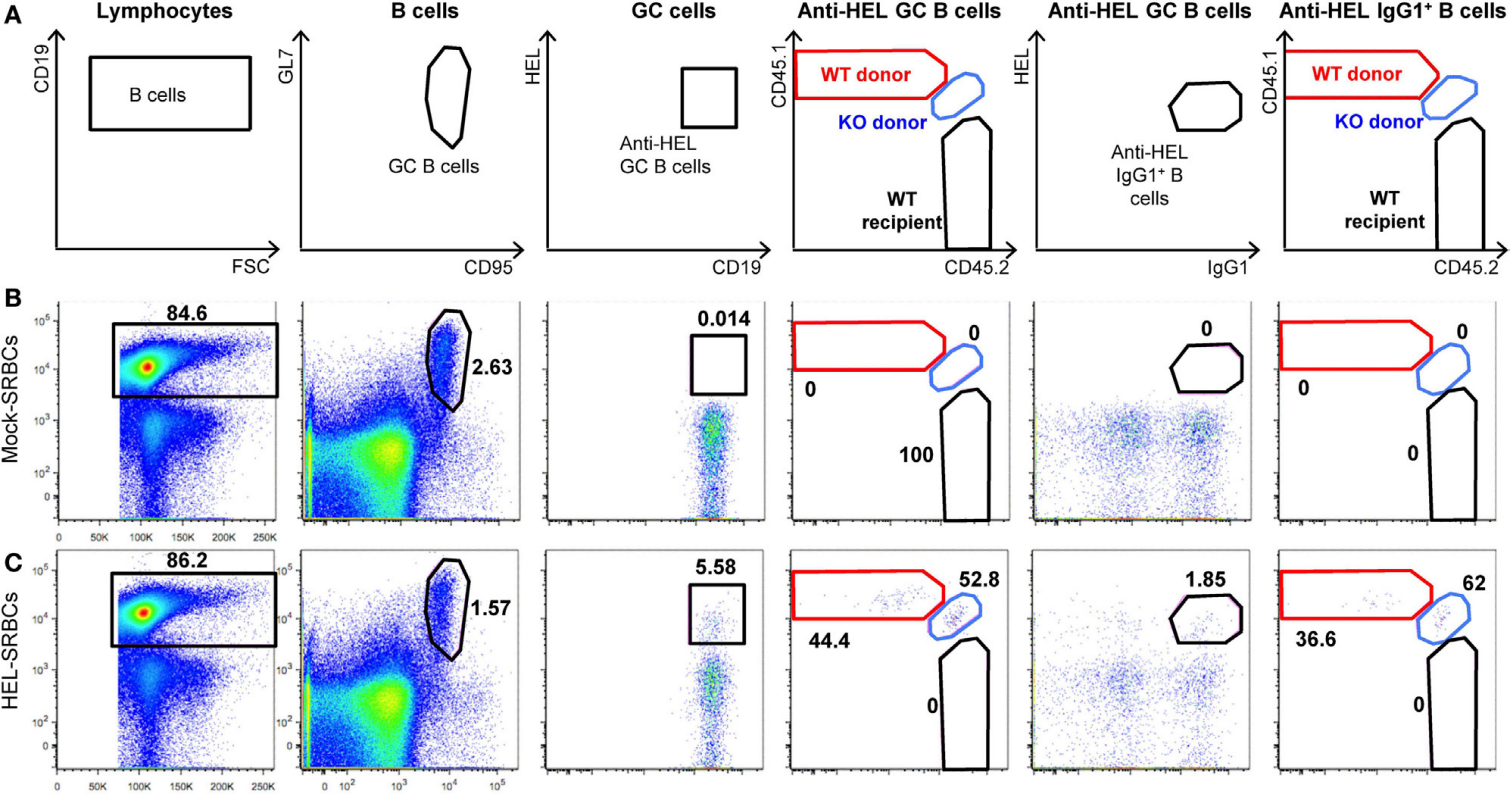
***Slc1a5^−/−^* B cells generate a functional germinal center (GC) response**. **(A)** Representative gating strategy for hen egg lysozyme (HEL)-binding GC B cells in the spleen and representative flow cytometry dot plots for mice immunized with sheep red blood cells (SRBCs) that were either unconjugated **(B)** or conjugated to HEL **(C)**. *n* = 8 mice injected with *Slc1a5^+/+^* CD45.1^+^ and *Slc1a5^−/−^* CD45.1^+^/CD45.2^+^ SW_HEL_ B cells and SRBC-HEL. *n* = 2 Mock-SRBC control mice injected with *Slc1a5^+/+^* CD45.1^+^ and *Slc1a5^−/−^* CD45.1^+^/CD45.2^+^ SW_HEL_ B cells and unconjugated SRBCs.

To analyze immune cell function, *Slc1a5^−/−^* and *Slc1a5^+/+^* mice were immunized with inactivated BP and alum-precipitated chicken gamma globulin (CGG), coupled to the hapten azo-benzene-arsonate (ABA). Both treatments resulted in similar primary and secondary antibody responses in wild-type and *Slc1a5^−/−^* mice (Figure 7). This indicates that antibody responses were unaffected by SLC1A5 deficiency. Finally, to further characterize *Slc1a5^−/−^* B cell effector function, we transferred approximately 6.5 × 10^4^ SW_HEL_ wild-type (expressing CD45.1) and 6.5 × 10^4^ *Slc1a5^−/−^* B cells (expressing CD45.1 and CD45.2; 1:1 ratio) into C57BL/6 mice and at the same time immunized them with sheep red blood cells conjugated to HEL. Analysis of B cell populations in the spleen 7 days post-immunization and adoptive transfer showed that *Slc1a5^−/−^* B cells were able to generate a functional germinal center (GC) response even when competing with WT cells (Figure 8). Thus, not only were B and T cell development unaffected in *Slc1a5^−/−^* mice, their B cell effector function was also normal.

## Discussion

Metabolic rewiring is now recognized as a hallmark of cancer (44). More specifically, glutamine addiction has been identified as a potential target to treat cancer metabolism either through inhibition of enzymes in the glutaminolysis pathway or its associated glutamine transporters (12, 45, 46). Due to the upregulation of SLC1A5 and LAT1 in a wide spectrum of human cancers (16), both transporters are currently being investigated as therapeutic targets (20, 24, 28, 47, 48).

However, glutamine is also crucial to the immune system, both to terminally differentiated immune cells such as neutrophils (49) and macrophages (49–51), and to activated lymphocytes (30, 31). Despite this, studies on the role of SLC1A5 in immune cells, which are themselves crucial to combating cancer, have only recently begun. In C57BL/6 × 129/sv mice, Nakaya et al. recently showed that SLC1A5 plays a role in CD4 helper T cell differentiation, *via* integration of TCR signaling through mTOR (32), possibly by providing an exchange substrate for leucine import by LAT1. Furthermore, SLC1A5 played a role in experimental allergic encephalomyelitis (EAE), a murine model of T cell-mediated autoimmunity (32). The study showed an effect of SLC1A5 deficiency on T cell development in 5-month-old C57BL/6 × 129/sv mice, but only mild effects in mice 6–7 weeks old, with reduced B cell numbers in the spleen (32). In line with these results, our investigation in mice on a pure C57BL/6 background showed no effect of SLC1A5 deficiency on T cell development in the spleen and thymus of 7–14-week-old mice (Figures 4 and 6). However, we also found no effect of SLC1A5 deficiency on B cell numbers in the spleen of these mice (Figure 5). This difference may be due to the different background of mice used in the two studies.

Moreover, our in depth characterization of B cell development in the bone marrow and spleen showed no differences in B cell progenitors and effector subsets between *Slc1a5^+/+^* and *Slc1a5^−/−^* mice. Even in the sensitive and competitive *in vivo* environment of mixed bone marrow chimeras, *Slc1a5^−/−^* and wild-type HSCs demonstrated the same capacity to reconstitute the B and T cell compartments. The lack of effect of SLC1A5 deficiency on HSC development in the bone marrow is in line with reports demonstrating that long-term (LT) HSCs survive in a highly hypoxic environment and are characterized by strong *glycolytic* metabolism and upregulation of *Hif-1*α (52). These results indicate either that the function of SLC1A5 is redundant in developing lymphocytes, and/or that SLC1A5 deficiency can be compensated for by upregulation of other transporters. This is similar to the scenario described by Broer et al. (9) in 143B human osteosarcoma cells where net glutamine import is mediated in large part not by SLC1A5, but by the sodium neutral amino acid transporters SNAT1 and SNAT2 (9). Moreover, MCF-7 breast cancer cells are also resistant to silencing of SLC1A5 (28). In these cell lines, SLC1A5 is not required for net uptake of glutamine, but rather for avoiding an amino acid starvation response. This is in line with the earlier finding by Bode et al. (1) that competitive inhibition of SLC1A5-mediated glutamine uptake in human hepatoma cell lines blocked their proliferation only in cells lacking expression of SNAT1 and SNAT2, and exhibiting low mRNA levels of glutamine synthetase (1). Indeed, we showed here that despite the dramatic increase in glutamine flux across the plasma membrane observed in LPS-activated relative to resting lymphocytes, B and T cell proliferation was only transiently affected by SLC1A5 deficiency, and only under strong stimulus conditions. This finding, together with the findings of Bode et al. (1) and Broer et al. (9), indicates that as in 143B cells, MCF-7 cells, and the hepatoma cell lines HepG2 and Hep3B, SLC1A5 may act as an “amino acid harmonizer” in lymphocytes, while other transporters such as SNAT1 (SLC38A1) and SNAT2 (SLC38A2) mediate net import of glutamine. This is supported by gene expression data in the Immgen database (53) showing high expression, particularly of SLC38A2 across the immune system. B cells undergo phases of intense proliferation, but most likely under nutrient-replete conditions. Under these conditions, lack of SLC1A5 may not have a strong impact.

Nakaya et al. (32) demonstrated an effect of SLC1A5 deficiency on T cell effector function, with impaired differentiation of helper T cells toward the Th1 and Th17 subsets. The strong utilization of glutamine by activated lymphocytes is well documented (4–6, 30, 54, 55), as is the importance of glutamine to T cell differentiation and effector function (30, 31, 56, 57). However, the role of glutamine is relatively uncharacterized in B cells, and the role of SLC1A5 in B cell development and function is even less well known. Our findings that *Slc1a5^−/−^* mice elicit normal primary and secondary antibody responses to immunization and that *Slc1a5^−/−^* B cells are capable of generating a normal GC response (even when competing with wild-type B cells) indicate that B cell effector function is not significantly affected by SLC1A5 deficiency. We also did not find a reduction in the number of B cells in the spleen previously reported by Nakaya et al. (32). A likely explanation for this difference is the use of mice from a mixed 129/C57BL/6 background by Nakaya et al. and mice on a pure C57BL/6N background in our study.

In summary, our data indicate a significant but not exclusive role for SLC1A5 in glutamine uptake by activated lymphocytes, but a role that is neither indispensable to proliferation in response to mitogen stimulation, nor crucial to B and T cell development and B cell effector responses, in C57BL/6 mice. These results suggest that treatment of cancers with SLC1A5 inhibitors may only have a limited impact on the adaptive immune system.

## Ethics Statement

All animal procedures for this study were approved by the ANU Animal Ethics and Experimentation Committee under protocol A2014/62.

## Author Contributions

EM-F, AB, and MY performed experiments. AE and SB devised the study. EM-F, AB, AE, and SB contributed to data analysis and interpretations. EM-F, AE, and SB wrote the manuscript. All the authors approved the final version of the manuscript.

## Conflict of Interest Statement

The authors declare that the research was conducted in the absence of any commercial or financial relationships that could be construed as a potential conflict of interest.

## Supplementary Material

Figure S1 **Normal B cell reconstitution in mixed BM chimeras**. We generated mixed BM chimeras by injecting a mix of CD45.1^+^ WT BM and CD45.2^+^ Slc1a5*^−/−^* bone marrow into sublethally irradiated CD45.2^+^ Rag1*^−/−^* recipients. As controls, we also used a mix of CD45.1^+^ WT with CD45.2^+^ WT BM cells. To test the efficiency of B cell reconstitution, we normalized the percentage of CD45.2^+^ B cells to the percentage of CD45.2^+^ T cells in the same mouse. Statistical analysis was done using a Student’s *t*-test. No statistical significant difference was found between the two sets of chimeras.
