# Supplementary figures and images for: ASCT2 (SLC1A5)-Deficient Mice Have Normal B-Cell Development, Proliferation, and Antibody Production
Source: Front Immunol. 2017 May 12;8:549. doi: 10.3389/fimmu.2017.00549 (PMC5427077; doi:10.3389/fimmu.2017.00549)

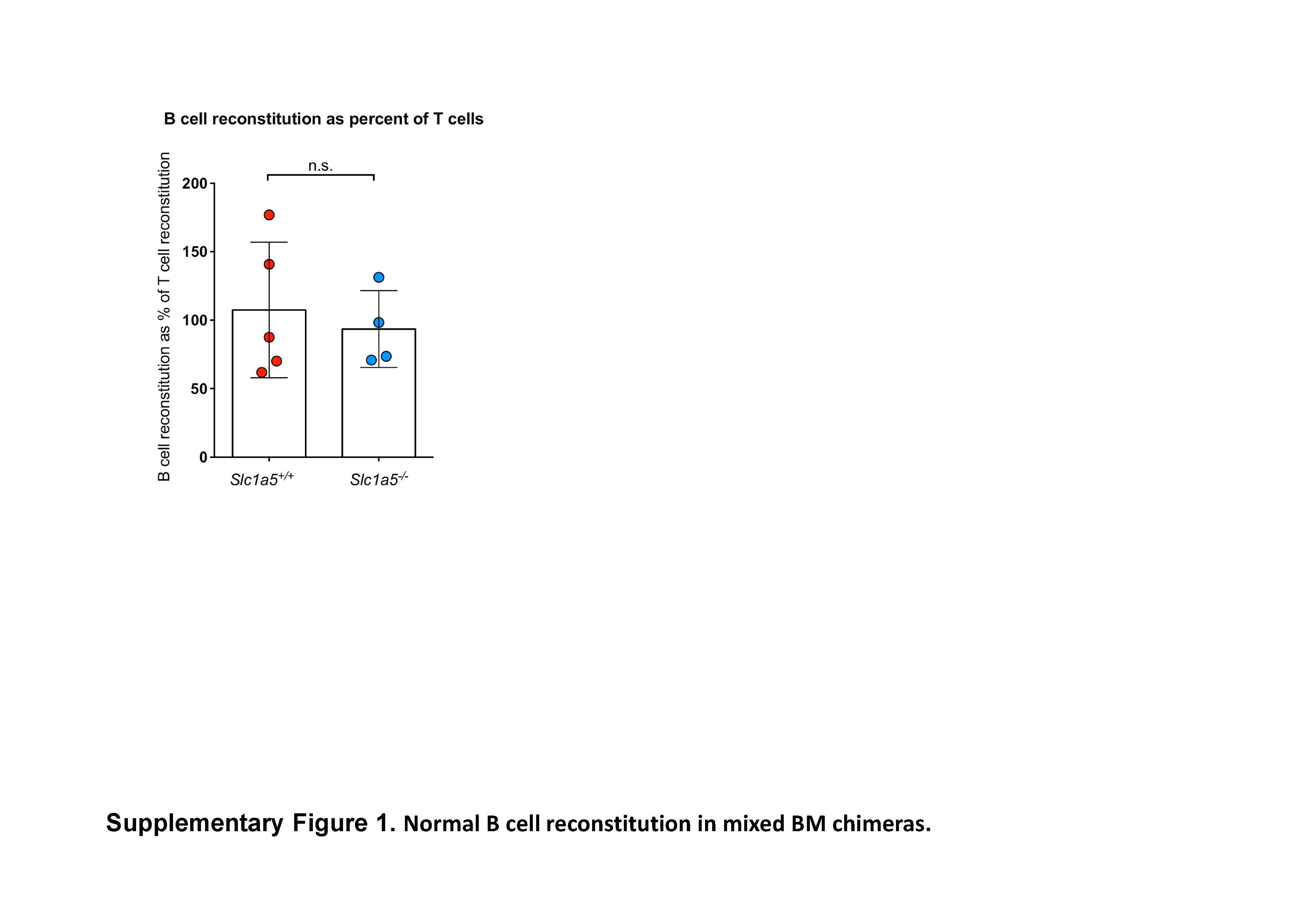

Supplement: Figure S1 — Normal B cell reconstitution in mixed BM chimeras. We generated mixed BM chimeras by injecting a mix of CD45.1+ WT BM and CD45.2+ Slc1a5−/− bone marrow into sublethally irradiated CD45.2+ Rag1−/− recipients. As controls, we also used a mix of CD45.1+ WT with CD45.2+ WT BM cells. To test the efficiency of B cell reconstitution, we normalized the percentage of CD45.2+ B cells to the percentage of CD45.2+ T cells in the same mouse. Statistical analysis was done using a Student’s t-test. No statistical significant difference was found between the two sets of chimeras. [file image_1.jpeg]
